# Supplementary material for: Venomix: a simple bioinformatic pipeline for identifying and characterizing toxin gene candidates from transcriptomic data
Source: PeerJ. 2018 Jul 31;6:e5361. doi: 10.7717/peerj.5361 (PMC6074769; doi:10.7717/peerj.5361)
Supplement: Supplemental Information 3 [file peerj-06-5361-s003.gz › FinalOutput_E-6/Acidic_phospholipase_A2_1_322/finaltree.pdf]

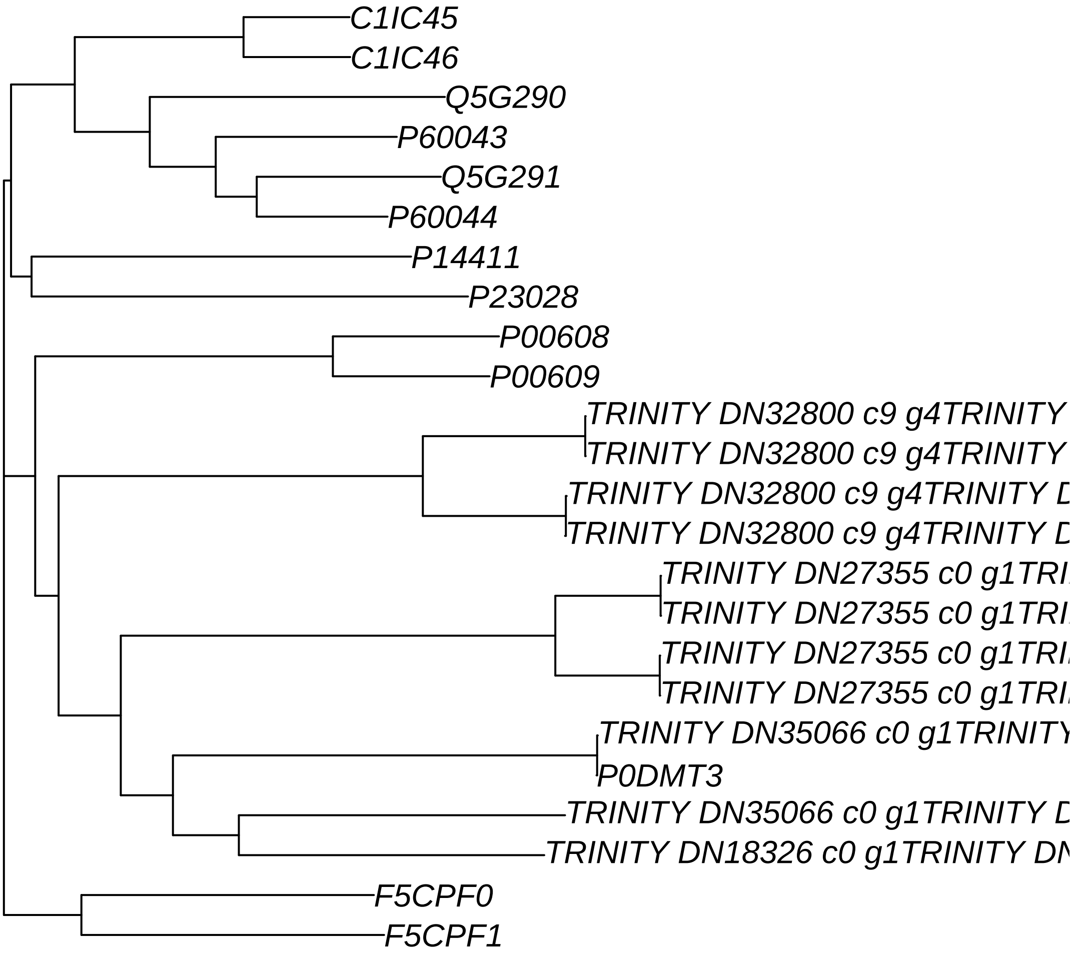
C1IC45
C1IC46
Q5G290
P60043
Q5G291
P60044
P14411
P23028
P00608
P00609
TRINITY DN32800 c9 g4TRINITY
TRINITY DN32800 c9 g4TRINITY
TRINITY DN32800 c9 g4TRINITY D
TRINITY DN32800 c9 g4TRINITY D
TRINITY DN27355 c0 g1TRI
TRINITY DN27355 c0 g1TRI
TRINITY DN27355 c0 g1TRI
TRINITY DN27355 c0 g1TRI
TRINITY DN35066 c0 g1TRINITY
P0DMT3
TRINITY DN35066 c0 g1TRINITY D
TRINITY DN18326 c0 g1TRINITY D
F5CPF0
F5CPF1
